# Factors influencing the uptake of intermittent preventive treatment among pregnant women in sub-Saharan Africa: a multilevel analysis

**DOI:** 10.1186/s13690-021-00707-z

**Published:** 2021-10-21

**Authors:** Eugene Kofuor Maafo Darteh, Kwamena Sekyi Dickson, Bright Opoku Ahinkorah, Bernard Afriyie Owusu, Joshua Okyere, Tarif Salihu, Vincent Bio Bediako, Eugene Budu, Wonder Agbemavi, Jane Odurowaah Edjah, Abdul-Aziz Seidu

**Affiliations:** 1 Department of Population and Health, University of Cape Coast, Cape Coast, Ghana; 2 School of Public Health, Faculty of Health, University of Technology Sydney, Sydney, Australia; 3 Guidance and Counselling Centre, University of Cape Coast, Cape Coast, Ghana; 4 Department of Estate management, Takoradi Technical University, Takoradi, Ghana

**Keywords:** Malaria in pregnancy, Malaria Indicator survey, IPTp-SP, Sub-Saharan Africa

## Abstract

**Background:**

Intermittent Preventive Treatment (IPT) of malaria in pregnancy is a full therapeutic course of antimalarial sulfadoxine-pyrimethamine (SP) medicine given to pregnant women in their second trimester at routine antenatal care visits, regardless of whether the recipient is infected with malaria. Given the negative consequences of malaria in pregnancy, studies on Intermittent Preventive Therapy with Sulfadoxine-Pyrimethamine (IPTp-SP) are important benchmarks for understanding the extent of malaria control and prevention during pregnancy. We, therefore, examined the factors associated with the uptake of IPTp-SP among pregnant women in sub-Saharan Africa.

**Methods:**

We used data from the current versions of the Malaria Indicators Survey of 12 countries in sub-Saharan Africa. Women aged 15–49 years participated in the surveys. The analyses were carried out using Stata version 14.2. Descriptive (frequencies and percentages) and multilevel regression analyses were carried out. The results of the multilevel regression analysis were presented as adjusted odds ratios (aOR) with 95% confidence intervals (CIs).

**Results:**

The average prevalence of uptake of IPTp-SP among pregnant women in the studied sub-Saharan African countries was 30.69%, with the highest and lowest prevalences in Ghana (59.64%) and Madagascar (10.08%), respectively. Women aged 40–44 compared to those aged 15–19 (aOR = 1.147, 95%CI = [1.02,1.30) had higher odds of receiving 3 or more doses of IPTp-SP. Women with a secondary/higher level of education compared to those with no formal education (aOR = 1.12, 95%CI = 1.04,1.20] also had higher odds of receiving 3 or more doses of IPTp-SP. Women who were exposed to malaria messages on the radio (aOR = 1.07, 95%CI = 1.02,1.12] and television (aOR = 1.13,95%CI = [1.05,1.21]) had higher odds of receiving 3 or more doses of IPTp-SP compared to those who were not exposed.

**Conclusion:**

Our study indicates that the uptake of IPTp-SP is relatively low among the countries included in this study, with significant inter-country variations. Higher educational level, exposure to media, low parity, and higher age group were associated with higher odds of optimal IPTp-SP uptake. National policies, programs, guidance services such as information service and counselling and other interventions aimed at improving the coverage and uptake of IPTp-SP must be targeted at women with low level of education, non-exposure to media, high parity, and younger age group to attain the desired outcome.

**Supplementary Information:**

The online version contains supplementary material available at 10.1186/s13690-021-00707-z.

## Background

Malaria among pregnant women is a major public health concern particularly in sub-Saharan Africa (SSA) where malaria exerts the highest health and socio-economic burden. The main infection parasite *plasmodium falciparum* is responsible for 99% of all malaria cases during pregnancy [1]. In 2018, about 29% (over 11 million) of all malaria cases occurred among pregnant women with most cases in SSA – West and Central Africa (35%), followed by East and Southern Africa (20%). Out of the over 38 million pregnancies that occurred in SSA in 2018, over 5.5 million children had low birth rates out of which 872,000 were due to malaria infection [2]. About 70% of all malaria deaths were recorded among pregnant women and their children under age 5. The WHO recommends in addition to vector control, and prompt diagnosis and effective treatment of malaria, intermittent preventive treatment of malaria with sulfadoxine-pyrimethamine (IPTp-SP) - the only antimalarial currently recommended for malaria-endemic areas in Africa [2].

Intermittent Preventive Treatment of malaria in pregnancy (IPTp) is a full therapeutic course of antimalarial Sulfadoxine-Pyrimethamine (SP) medicine given to pregnant women in their second trimester at routine antenatal care visits, regardless of whether the recipient is infected with malaria [3]. In addition to the several maternal morbidities including anemia, infants born to mothers with untreated malaria have reduced immunity to malaria, and are at increased risk of placental malaria, rapid malaria progression, and death [2, 4]. To combat the burden of malaria on pregnant women and their children in moderate to high transmission areas in SSA, a minimum of three (3) instead of two (2) doses of SP during pregnancy within 1 month apart is recommended [2]. IPTp-SP has several benefits for the mother and her unborn child. These include reducing maternal and fetal anemia, placental parasitemia, low birth weight, and neonatal mortality [2, 5, 6]. The objectives of reducing and eliminating the malaria burden are intrinsically linked to most of the Sustainable Development Goals (SDGs) and are central to SDG 3 which seeks to ensure healthy lives and promote well-being for all at all ages.

Unlike all other parts of Africa, countries in North Africa such as Morocco in 2010, and Algeria in 2019 have eliminated malaria, and other countries such as Egypt have attained 3 consecutive years of zero non-indigenous cases [2]. However, malaria is still highly endemic in SSA [2]. For instance, over 50% of all malaria cases occurred in countries such as Nigeria (25%), DR Congo (12%), Uganda (5%), Cote d’Ivoire (4%), Mozambique (4%), and Niger (4%). In Ghana and Nigeria for instance, notable cases of increase in malaria (8 and 6% respectively) were recorded in 2018 [2]. The WHO revised the IPTp-SP policy in 2012 and recommended that all areas with moderate to high malaria transmission in Africa should make efforts to increase access to three doses of IPTp-SP.

Since then, several countries have revised their IPTp-SP policy to reflect the new three doses recommendation. For instance, Ghana was the first country in SSA to revise its policy in 2012. Other countries such as Angola, DR Congo, Guinea, Malawi, and Senegal revised their policy in 2013, and most countries adopted the three doses of IPTp-SP policy in 2014 [7]. However, progress towards improving the three doses IPTp-SP uptake among pregnant women has been inadequate [8–10] and country and regional variations have been recorded as well [9–11]. Andrews et al. [9] for instance recommended further research into the factors responsible for such discrepancies in IPTp-SP uptake across countries.

Several studies have been conducted on IPTp-SP across various parts of Africa. Yaya and his colleagues [12] in their studyacross eight sub-Saharan African countries reported that the prevalence of IPTp-SP was highest in Ghana, Kenya, and Sierra Leone. They found that women from middle to richest households and those with lower levels of education had higher proportions of taking the recommended three doses of IPTp-SP. Evidence from various countries in SSA have also shown significant relationships between socio-economic characteristics and IPTp-SP uptake. For instance, the odds of IPTp-SP uptake are likely to be higher among women who utilize ANC services [9, 13]. Other factors that influence the uptake of IPTp-SP include education, occupation, marital status, ethnicity, exposure to IPTp-SP messages, and parity [11, 14, 15]. Given the negative consequences of malaria in pregnancy [2], studies on IPTp-SP are important benchmarks for understanding the extent of malaria control and prevention during pregnancy.

With the availability of recent datasets across countries in SSA, where the prevalence of malaria is predominant, it is essential that research is carried out on the uptake of IPTp-SP using these recent datasets to understand if the phenomenon has changed over time, in terms of prevalence and predictors. We, therefore, examined the predictors of IPTp-SP uptake across 12 countries in SSA. Our study adds to the existing literature on IPTp-SP in SSA, and differs from the study of Yaya et al. [12], by considering recent data from the malaria indicators survey (MIS) of 12 countries in SSA ranging from 2015 to 2019. Knowing the predictors of IPTp-SP uptake is critical to identifying areas where national and international interventions could yield greater cost-effective results towards the upscale of IPTp-SP treatment and mitigate the increased burden of malaria on pregnant women and their children living in moderate to high malaria-prone areas in SSA.

## Methods

### Data source

Data were sourced from the recent version of the MIS of 12 countries in SSA from 2015 to 2019. The countries included were those that had data on IPTp-SP as well as the explanatory variables considered in our study. The Surveys were conducted by technical assistance as well as funding by Inner City Fund International through the Demographic and Health Survey (DHS) Program, which is a USAID-funded project providing support and technical assistance in the implementation of population and health surveys in countries worldwide. The stratified two-stage cluster sample is used by MIS surveys to sample women aged 15–49. Sampling strata, from which clusters are selected per stratum by a probability-proportional-to-size selection, were generated in the first step. A full listing of the selected clusters functions as the second stage sampling frame. In the second level, by equal probability systematic sampling, households are selected from each cluster. The surveys aimed to provide quality data to evaluate progress towards selected goals and objectives needed for successful monitoring and to assess the implementation and measurement of the national malaria programs. Specifically, the surveys aimed to assess the measures of possession and usage of mosquito bed nets; coverage of the sporadic preventive care program for pregnant women; behaviour seeking treatment; assessment of awareness, behaviours, and behavioural indicators; related to malaria control; and the determination of malaria and anaemia-related factors. In addition to these issues, several other questions are answered about basic demographics, education, the use of ITNs, prevention of malaria during pregnancy, and malaria awareness. Women aged 15–49 (*n* = 43,961) with complete information on all variables considered in the current study participated in the study. The dataset is freely available for download at https://dhsprogram.com/data/available-datasets.cfm

### Variables for the study

#### Outcome variable

The outcome variable was the use of IPTp-SP. It was assessed by asking the respondents whether or not they took Fansidar (sulfadoxine-pyrimethamine, SP) during their last pregnancy, and how many times it was taken. IPTp-SP administration is considered to be one of the three main strategies recommended by the WHO for the management of malaria in pregnancy in secure areas of transmission of malaria during antenatal visits [16]. As the WHO recommends at least three doses of SP, we used the guideline of three doses for the current study to classify the variable into two; incomplete (0–2 doses) and complete (3 or more doses) [11, 12, 16].

#### Independent variables

The independent variables were grouped into individual and contextual variables. The individual level variables comprised age (15–19, 20–24, 25–29, 30–34, 35–39, 40–44, 45–49), educational level (no formal education, primary, secondary/higher), parity (1–3, 4 or more), received malaria information on television (Yes, No), and received malaria information on the radio (Yes, No) (See Table 1). The contextual level variables included place of residence (rural, urban), wealth status (poorest, poorer, middle, richer, richest) and sex of household head (male, female). These variables were chosen based on their association with IPTp-SP in previous studies [11, 12, 14].

**Table 1:** Prevalence of IPTp-SP in last pregnancy among women in sub-Saharan Africa (n = 43,961)

Variables	Weighted N	Weighted %	Prevalence of IPTp-SP	
			0-2 doses	3 or more doses
**Individual level factors**				
**Age**				
15–19	4022	9.15	72.52	27.48
20–24	10,457	23.79	69.53	30.47
25–29	10,912	24.82	68.52	31.48
30–34	8634	19.64	69.73	30.27
35–39	6125	13.93	68.07	31.93
40–44	2833	6.45	67.73	32.27
45–49	977	2.22	71.21	28.79
**Education**				
No formal education	16,801	38.22	68.82	31.18
Primary	15,849	36.05	70.97	29.03
Secondary/Higher	11,311	25.73	67.72	32.28
**Parity**				
1 to 3	24,626	56.02	68.93	31.07
4 or more	19,335	43.98	69.80	30.20
**Exposure to malaria information on Radio**				
No	19,754	44.94	69.97	30.03
Yes	24,207	55.06	68.77	31.23
**Exposure to malaria information on TV**				
No	31,981	72.75	70.43	29.57
Yes	11,980	27.25	66.33	33.67
**Contextual factors**				
**Residence**				
Urban	11,964	27.22	67.43	32.57
Rural	31,997	72.78	70.01	29.99
**Wealth status**				
Poorest	9451	21.5	70.75	29.25
Poorer	9352	21.27	69.47	30.53
Middle	8622	19.61	69.05	30.95
Richer	8414	19.14	68.29	31.71
Richest	8122	18.48	68.79	31.21
**Sex of Household Head**				
Male	35,628	81.04	69.23	30.77
Female	8333	18.96	69.64	30.36
**Country**				
Burkina	4423	10.06	42.54	57.46
Ghana	2161	4.91	40.36	59.64
Kenya	2232	5.08	78.05	21.95
Liberia	2126	4.84	76.43	23.57
Madagascar	5092	11.58	89.92	10.08
Mali	5054	11.5	77.74	22.26
Malawi	2102	4.78	65.35	34.65
Mozambique	3288	7.48	58.09	41.91
Nigeria	3923	8.92	79.18	20.82
Sierra Leone	4457	10.14	69.39	30.61
Tanzania	5036	11.46	77.48	22.52
Uganda	4068	9.25	60.41	39.59
**All countries**	43,961	100.0	69.31	30.69

### Statistical analyses

All the analyses were carried out in Stata version 14.2 in two main ways using descriptive and inferential techniques. First, the data were extracted and pooled. Afterwards, frequencies and percentages were used to describe the sample and present the prevalence of IPTp-SP uptake. Next,  we fitted a two-level multivariable multilevel logistic regression models to analyse the association between individual and contextual factors associated with IPTp-SP. This was done to cater for the clustering in the MIS dataset. The two-level modelling in this study implies that women were nested within households and households were nested within clusters. Clusters were considered as random effects to cater for the unexplained variability at the contextual level. The multilevel models had both fixed and random effects. The results of the fixed effects analysis were presented as adjusted odds ratio (aOR) with their corresponding 95% confidence intervals (CI) whereas the random effects were assessed with intra-cluster correlation (ICC). Model comparison was done using the log-likelihood ratio (LLR) and Akaike’s Information Criterion (AIC) tests. We checked for multicollinearity among the variables by using variance inflation factor (VIF) and there was no evidence of multicollinearity (mean VIF = 1.46). We applied the inherent sample weight (v005/1000000) and the survey (svy) command in Stata version 14.2 to adjust for the complex sampling structure of the data.

### Ethical approval

The research was based on an analysis of anonymized secondary data sources in the DHS public domain. Therefore, no further approval was expected. That being said, the authors gained permission for the reuse of the data.

## Results

### Prevalence of IPTp-SP in last pregnancy among women in sub-Saharan Africa

Table 1 presents results on the prevalence of IPTp-SP in last pregnancy among women in SSA by their socio-demographic characteristics. In terms of the WHO recommended number of doses (3 or more doses), the average prevalence among women in the studied sub-Saharan African countries were 30.69%, with the highest and lowest prevalences in Ghana (59.64%) and Madagascar (10.08%), respectively. Across the socio-demographic characteristics of women, the highest prevalence was observed among women aged 40–44 (32.27%), women with secondary/higher education (32.28%), those in urban areas (32.57%), richer women (31.71%), women with parity 1–3 (31.07%), women in male-headed households (30.77%), women who were exposed to the radio (31.23%) and women who were exposed to television (33.67%).

### Factors associated with IPTp-SP uptake in last pregnancy among women in sub-Saharan Africa

#### Fixed effects (measure of association) results

Table 2 shows the results of a multilevel logistic regression analysis on the factors associated with IPTp-SP uptake in last pregnancy among women in SSAa. At the individual level, we found that women aged 40–44 compared to those aged 15–19 (aOR = 1.147, 95%CI = [1.02,1.30) had higher odds of receiving 3 or more doses of IPTp-SP. Women with a secondary/higher level of education compared to those with no formal education (aOR = 1.12, 95%CI = 1.04,1.20] also had higher odds of receiving 3 or more doses of IPTp-SP. Women who were exposed to malaria messages on the radio (aOR = 1.07, 95%CI = 1.02,1.12] and television (aOR = 1.13,95%CI = [1.05,1.21]) had higher odds receiving 3 or more doses of IPTp-SP compared to those who were not exposed. At the contextual level, compared to women in Ghana, all other countries had lower odds of receiving 3 or more doses of IPTp-SP. Country specific results have been presented as supplementary files for all the 12 countries (Table S1, S2, S3, S4, S5, S6, S7, S8, S9, S10, S11 and S12).

**Table 2:** Multi-level regression analysis on predictors of optimal IPTp-SP in last pregnancy among women in sub-Saharan Africa

Variables	Model 0	Model 1	Model 2	Model 3
		aOR[95%CI]	aOR[95%CI]	aOR[95%CI]
**Individual level factors**				
**Age**				
15–19		[1.00,1.00]		[1.00,1.00]
20–24		1.09*[1.01,1.18]		1.03 [0.95,1.13]
25–29		1.12**[1.03,1.22]		1.049 [0.96,1.15]
30–34		1.15** [1.05,1.26]		1.075 [0.98,1.18]
35–39		1.21***[1.09,1.33]		1.100 [0.99,1.22]
40–44		1.25***[1.12,1.41]		1.147*[1.01,1.30]
45–49		1.171 [1.00,1.38]		1.105 [0.93,1.31]
**Education**				
No formal education		[1.00,1.00]		[1.00,1.00]
Primary		0.90***[0.85,0.94]		1.07* [1.01,1.13]
Secondary/Higher		1.00 [0.94,1.06]		1.12**[1.04,1.20]
**Parity**				
1 to 3		1.11***[1.05,1.17]		1.11***[1.04,1.17]
4 or more		Ref		Ref
**Exposure to malaria information on Radio**				
No		[1.00,1.00]		[1.00,1.00]
Yes		1.03 [0.99,1.08]		1.068**[1.02,1.12]
**Exposure to malaria information on TV**				
No		[1.00,1.00]		[1.00,1.00]
Yes		1.174***[1.11,1.24]		1.128*** [1.05,1.21]
**Contextual factors**				
**Place of Residence**				
Urban			[1.00,1.00]	[1.00,1.00]
Rural			0.948 [0.89,1.01]	0.955 [0.90,1.02]
**Wealth status**				
Poorest			[1.00,1.00]	[1.00,1.00]
Poorer			1.058 [0.99,1.13]	1.028 [0.96,1.10]
Middle			1.078*[1.01,1.15]	1.019 [0.95,1.09]
Richer			1.093*[1.02,1.17]	0.98 [0.91,1.06]
Richest			1.111*[1.02,1.21]	0.91 [0.82,1.01]
**Sex of Household Head**				
Male			[1.00,1.00]	[1.00,1.00]
Female			0.981 [0.93,1.04]	0.98 [0.93,1.04]
**Country**				
Burkina			0.821***[0.74,0.92]	0.921 [0.82,1.03]
Ghana			[1.00,1.00]	[1.00,1.00]
Kenya			0.193*** [0.17,0.22]	0.202***[0.18,0.23]
Liberia			0.207***[0.18,0.24	0.225***[0.20,0.26]
Madagascar			0.0719***[0.06,0.08]	0.0779*** [0.07,0.09]
Mali			0.180***[0.16,0.20]	0.196***[0.17,0.22]
Malawi			0.326*** [0.29,0.37]	0.363***[0.32,0.42]
Mozambique			0.510***[0.45,0.58]	0.557***[0.49,0.63]
Nigeria			0.182***[0.16,0.20]	0.192***[0.17,0.22]
Sierra Leone			0.294***[0.26,0.33]	0.327*** [0.29,0.37]
Tanzania			0.157*** [0.14,0.18]	0.171***[0.15,0.19]
Uganda			0.424*** [0.38,0.47]	0.458***[0.41,0.51]
**Random effects**				
PSU variance (95% CI)	0.246 (0.19–0.31)	0.234 (0.19–0.30)	0.092 (0.07–0.12)	0.013 (0.07–0.12)
ICC	0.069	0.066	0.0273	0.0273
Wald chi-square and *p*-value	Ref	120.31	3839.66	3875.83
LR Test	490.76	470.73	219.04	218.29
**Model fitness**				
Log-likelihood	−27,117.179	−27,057.15	−24,992.34	−24,966.9
AIC	54,238.36	54,140.3	50,022.68	49,993.79
PSU	584	584	584	584
N	43,961	43,961	43,961	43,961

Exponentiated coefficients; 95% confidence intervals in brackets

Model 0 is the null model, a baseline model without any determinant variable

Model 1 = Individual level variables

Model 2 = Community level variables

Model 3 is the final model adjusted for individual and household/community level variables

^*^ *p* < 0.05, ^**^ *p* < 0.01, ^***^ *p* < 0.001, [1.00,1.00] = Ref; *PSU* Primary Sampling Unit, *ICC* Intra-Class Correlation, *LR Test* Likelihood ratio Test, *AIC* Akaike’s Information Criterion

#### Random effects (measures of variations) results

The empty model (Model 0), as shown below in Table 2, depicted a substantial variation in the likelihood IPTp-sp in sub-Saharan Africa across the Primary Sampling Units (PSUs) clustering [σ2 = 0.25; 95%(CI = 0.19–0.31]. The Model 0 indicated that 10% of the variation in IPTp-SP in SSA was attributed to the variation between the clusters (ICC = 0.10). At the individual level model (model 1), the ICC remained 10%. In the household/community-level only (Model 2), the ICC declined to 3%. This remained again in the complete model with both the individual and contextual factors (Model 3). The AIC in the complete model had the lowest value therefore this was selected to predict the likelihood of IPTp-Sp uptake among women in sub-Saharan Africa. Country specific results have been presented as supplementary files for all the 12 countries (Table S1, S2, S3, S4, S5, S6, S7, S8, S9, S10, S11 and S12).

## Discussion

In this study, we examined the predictors of IPTp-SP uptake across 12 countries in SSA. Generally, our findings showed that the prevalence of the uptake of IPTp-SP was low, with a little above one-quarter of pregnant women given IPTp-SP. Our result is consistent with previous related studies [11, 12, 14, 16] that also found low prevalence of the  uptake of IPTp-SP in SSA. This low prevalence of the uptake of IPTp-SP raises concern for malaria prevention and eradication in SSA. If countries continue on this trajectory, then it will be near impossible to achieve a 90% decline in new infections and mortality due to malaria by 2030 as stipulated by the WHO Global Technical Strategy for Malaria 2016–2030 [2].

Since the overall average for IPTp-SP was low, our findings indicate that there were inter-country variations in the uptake of IPTp-SP, with Ghana reporting the highest prevalence (59.64%) while Madagascar had the lowest prevalence. The high prevalence of IPTp-SP in Ghana affirms the findings of previous studies [11, 12, 14]. Ghana’s high prevalence of the uptake of IPTp-SP could be ascribed to the introduction and early approval of IPTp-SP as a nationwide medical measure in 2004 [12, 17]. A plausible explanation for Madagascar’s low prevalence in the uptake of IPTp-SP could be due to the critical role of the low supply in determining the extent of coverage [18].

As expected, a higher educational level was a protective factor against receiving no dose of IPTp-SP. Compared to women with no formal education, those with higher education had higher odds to report receiving 3 or more doses of IPTp-SP. The current finding is congruent with extant studies that have found IPTp-SP uptake, and dose completion to be much profound among women with higher education [19, 20]. This could be justified from the perspective that higher education empowers women, improves their decision-making prowess, and provides them with accurate information, thereby clearing all misconceptions and myths surrounding IPTp-SP use. 

Results from this study suggest that age is a significant associated factor for the uptake of IPTp-SP among pregnant women in SSA. The odds of receiving 3 or more doses of IPTp-SP increased significantly as women grew older. From our study, women aged 40–44 had higher odds of receiving 3 or more doses of IPTp-SP compared to younger women (15–19). This is in consonance with reports by Azizi et al. [21], who indicated that older pregnant women were at a higher relative risk to have received the recommended dose of IPTp-SP compared to those aged between 15 and 19. Likewise, a similar finding was reported by Kibusi, Kimunai, and Hines [22]. A possible justification for this finding may be that the differentials in the uptake of IPTp-SP by age signifies the diverse appreciation and understanding of health as women age. Moreover, the age cohort of 15–19 years falls within the period of adolescence where pregnancy is often unplanned. Hence, such girls are could be unprepared for pregnancy, and therefore, are most likely not to attend antenatal care, during which  IPTp-SP are given.

Our study also shows that women who were exposed to radio and television had higher odds of receiving 3 or more doses of IPTp-SP compared to those who were not exposed to any of these media sources.  This result is expected as exposure to both radio and television may offer pregnant women an opportune moment to grasps, and have access to unadulterated, accurate, and timely information concerning malaria preventive measures, including receiving IPTp-SP. In every health intervention, access to quality information cannot be understated. It is this information through traditional mediums like radio and television that misconceptions, stereotypic viewpoints, and myths are exhaustively cleared to propel, and psyche pregnant women to receive IPTp-SP, as stipulated by the international standards on the recommended dosage.

Finally, the findings of this study revealed that the number of children ever born to a woman was a significant predictor of the uptake of IPTp-SP, with women who had 1–3 children reporting higher odds of receiving 3 or more doses of IPTp-SP. This finding is in line with the study of Kibusi et al. [22] who found that women with their first or second birth were more likely to have a higher uptake of IPTp-SP.

### Strength and limitations

This study has been significant in extending the current discourse on pregnant women’s uptake of IPTp-SP. A significant strength of this study lies in the use of a large sample size and recent survey data that makes the findings emanating from this study generalizable to women 15–19 years within the studied countries. Again, disaggregating the results by individual countries provides a robust information to understand the phenomenon within the various countris. Also, our study has implications for policy and practice as it highlights the key protective factors and risk factors that affect pregnant women’s uptake of IPTp-SP. Notwithstanding, the present study was not without limitations, therefore, conclusions drawn from the findings must be done with caution. The study relied on a secondary data source and thus limited our control over the variables and measurements within the datasets. Also, due to the cross-sectional nature of the data used, causal inferences cannot be assumed; only associations can be made. The variable that measures the prevalence of the uptake of IPTp-SP was self- reported, and retrospective in nature, thereby exposing the subjects to recall bias. Yet, we hold that the strengths of this study are far-reaching compared to the limitations.

## Conclusion

 Our study indicates that the uptake of IPTp-SP is relatively low among the countries included in this study, with significant inter-country variations. Higher educational level, exposure to media, low parity, and higher age cohort were the protective factors of  optimal IPTp-SP uptake. Given the existence of these factors, it is important for national policies, programs, interventions aimed at improving the coverage and uptake of IPTp-SP, to devote priority attention to these peculiar issues to attain the desired outcome. Qualitative studies may be needed to explore how these factors influence the uptake of IPTp-SP in SSA. This will be needed to forge a deeper understanding of the nuances surrounding IPTp-SP uptake by pregnant women.

## Supplementary Information

**Additional file 1.**

## Data Availability

The dataset is freely available for download at https://dhsprogram.com/data/available-datasets.cfm

## Abbreviations

AIC: Akaike’s Information Criterion
ANC: Antenatal Care
aOR: Adjusted odds Ratio
DHS: Demographic and Health Survey
ICC: Intra-cluster correlation (ICC)
IPT: Intermittent Preventive Treatment
IPTp-SP: Intermittent Preventive Therapy with Sulfadoxine-Pyrimethamine
LLR: Log-likelihood ratio
MIS: Malaria Indicators Survey
SDGs: Sustainable Development Goals
SSA: Sub-Saharan Africa
VIF: Variance inflation factor
WHO: World Health Organisation
