# Supplementary material for: Factors influencing the uptake of intermittent preventive treatment among pregnant women in sub-Saharan Africa: a multilevel analysis
Source: Arch Public Health. 2021 Oct 21;79:182. doi: 10.1186/s13690-021-00707-z (PMC8529836; doi:10.1186/s13690-021-00707-z)
Supplement: Supplementary file 1 — Additional file 1. [file 13690_2021_707_MOESM1_ESM.docx]

**Table S1: Multi-level regression analysis on predictors of optimal IPTp-SP in last Pregnancy among women in Burkina Faso**

|  | Model 0 | **Model 1** | **Model 2** | **Model 3** |
| --- | --- | --- | --- | --- |
| **Variable** |  | **aOR[95%CI]** | **aOR[95%CI]** | **aOR[95%CI]** |
| **Age** |  |  |  |  |
| 15-19 |  | [1.00,1.00] |  | [1.00,1.00] |
| 20-24 |  | 0.881 |  | 0.88 |
|  |  | [0.66,1.18] |  | [0.66,1.18] |
| 25-29 |  | 1.014 |  | 1.012 |
|  |  | [0.76,1.36] |  | [0.75,1.36] |
| 30-34 |  | 0.971 |  | 0.975 |
|  |  | [0.70,1.34] |  | [0.70,1.35] |
| 35-39 |  | 1.058 |  | 1.065 |
|  |  | [0.75,1.49] |  | [0.75,1.51] |
| 40-44 |  | 1.375 |  | 1.388 |
|  |  | [0.92,2.05] |  | [0.93,2.07] |
| 45-49 |  | 0.893 |  | 0.891 |
|  |  | [0.54,1.48] |  | [0.54,1.48] |
| **Education** |  |  |  |  |
| No formal education |  | [1.00,1.00] |  | [1.00,1.00] |
| Primary |  | 0.922 |  | 0.925 |
|  |  | [0.76,1.12] |  | [0.76,1.13] |
| SHS/Tertiary |  | 0.895 |  | 0.914 |
|  |  | [0.71,1.13] |  | [0.72,1.16] |
| **Parity** |  |  |  |  |
| 1-3 |  | 1.027 |  | 1.034 |
|  |  | [0.85,1.24] |  | [0.86,1.25] |
| 4 or more |  | [1.00,1.00] |  | [1.00,1.00] |
| **Radio** |  |  |  |  |
| No |  | [1.00,1.00] |  | [1.00,1.00] |
| Yes |  | 1.027 |  | 1.033 |
|  |  | [0.89,1.18] |  | [0.89,1.20] |
| **TV** |  | 1 |  | 1 |
| No |  | [1.00,1.00] |  | [1.00,1.00] |
| Yes |  | 1.116 |  | 1.139 |
|  |  | [0.93,1.34] |  | [0.93,1.40] |
| **Place of Residence** | |  |  |  |
| Urban |  |  | [1.00,1.00] | [1.00,1.00] |
| Rural |  |  | 1.17 | 1.171 |
|  |  |  | [0.83,1.64] | [0.83,1.65] |
| **Wealth** |  |  | 1 | 1 |
| Poorest |  |  | [1.00,1.00] | [1.00,1.00] |
| Poorer |  |  | 0.987 | 0.993 |
|  |  |  | [0.80,1.21] | [0.81,1.22] |
| Middle |  |  | 1.049 | 1.036 |
|  |  |  | [0.85,1.30] | [0.83,1.29] |
| Richer |  |  | 0.953 | 0.907 |
|  |  |  | [0.77,1.18] | [0.72,1.15] |
| Richest |  |  | 1.138 | 1.06 |
|  |  |  | [0.85,1.53] | [0.76,1.47] |
| **Sex of household head** | |  |  |  |
| Male |  |  | [1.00,1.00] | [1.00,1.00] |
| Female |  |  | 0.838 | 0.862 |
|  |  |  | [0.62,1.13] | [0.64,1.16] |
| **Random effects** |  |  |  |  |
| PSU variance (95% CI) | 0.57(0.42-0.75) | 0.57(0.42-0.75) | 0.57(0.42-0.75) | 0.56(0.422-0.748) |
| ICC | 0.15 | 0.147 | 0.148 | 0.145 |
| Wald chi-square and p-value | Ref | 12.53(0.324) | 3.68(0.72) | 16.15(0.51) |
| LR Test | 237.06 | 233.37 | 234.42 | 229.65 |
| **Model fitness** |  |  |  |  |
| Log-likelihood | -2891.7082 | -2885.374 | -2889.869 | -2883.55 |
| AIC | 5787.416 | 5796.748 | 5795.739 | 5805.1 |
| N | 4423 | 4423 | 4423 | 4423 |

Exponentiated coefficients; 95% confidence intervals in brackets

^*^ *p* < 0.05, ^**^ *p* < 0.01, ^***^ *p* < 0.001 , [1.00,1.00]=Ref; PSU=Primary Sampling Unit

ICC = Intra-Class Correlation; LR Test= Likelihood ratio Test; AIC = Akaike’s Information Criterion

Model 0 is the null model, a baseline model without any determinant variable

Model 1 = Individual level variables

Model 2 = Community level variables

Model 3 is the final model adjusted for individual and household/community level variables

**Table S2: Multi-level regression analysis on predictors of optimal IPTp-SP in last Pregnancy among women in Ghana**

|  | **Model0** | **Model 1** | **Model 2** | **Model 3** |
| --- | --- | --- | --- | --- |
| **Variable** |  | **aOR[95%CI]** | **aOR[95%CI]** | **aOR[95%CI]** |
| **Age** |  |  |  |  |
| 15-19 |  | [1.00,1.00] |  | [1.00,1.00] |
| 20-24 |  | 1.183 |  | 1.182 |
|  |  | [0.76,1.84] |  | [0.76,1.84] |
| 25-29 |  | 2.146^***^ |  | 2.166^***^ |
|  |  | [1.38,3.33] |  | [1.39,3.37] |
| 30-34 |  | 2.022^**^ |  | 2.053^**^ |
|  |  | [1.28,3.20] |  | [1.29,3.28] |
| 35-39 |  | 2.346^***^ |  | 2.388^***^ |
|  |  | [1.44,3.84] |  | [1.45,3.93] |
| 40-44 |  | 2.652^***^ |  | 2.695^***^ |
|  |  | [1.53,4.60] |  | [1.54,4.71] |
| 45-49 |  | 1.696 |  | 1.69 |
|  |  | [0.84,3.42] |  | [0.83,3.43] |
| **Education** |  |  |  |  |
| No formal education |  | [1.00,1.00] |  | [1.00,1.00] |
| Primary |  | 0.903 |  | 0.954 |
|  |  | [0.68,1.20] |  | [0.72,1.27] |
| SHS/Tertiary |  | 1.161 |  | 1.299 |
|  |  | [0.89,1.52] |  | [0.98,1.72] |
| **Parity** |  |  |  |  |
| 1-3 |  | 1.355^*^ |  | 1.400^*^ |
|  |  | [1.05,1.75] |  | [1.08,1.82] |
| 4 or more |  | [1.00,1.00] |  | [1.00,1.00] |
| **Exposure to malaria information on Radio** |  |  |  |  |
| No |  | [1.00,1.00] |  | [1.00,1.00] |
| Yes |  | 1.063 |  | 1.046 |
|  |  | [0.88,1.29] |  | [0.86,1.28] |
| **Exposure to malaria information on TV** |  |  |  |  |
| No |  | [1.00,1.00] |  | [1.00,1.00] |
| Yes |  | 1.156 |  | 1.384^*^ |
|  |  | [0.93,1.44] |  | [1.06,1.80] |
| **Place of Residence** |  |  |  |  |
| Urban |  |  | [1.00,1.00] | [1.00,1.00] |
| Rural |  |  | 1.156 | 1.085 |
|  |  |  | [0.85,1.57] | [0.80,1.47] |
| **Wealth** |  |  |  |  |
| Poorest |  |  | [1.00,1.00] | [1.00,1.00] |
| Poorer |  |  | 0.885 | 0.745 |
|  |  |  | [0.66,1.19] | [0.55,1.02] |
| Middle |  |  | 0.909 | 0.615^*^ |
|  |  |  | [0.65,1.26] | [0.42,0.91] |
| Richer |  |  | 1.006 | 0.591^*^ |
|  |  |  | [0.69,1.46] | [0.38,0.93] |
| Richest |  |  | 1.442 | 0.706 |
|  |  |  | [0.95,2.19] | [0.42,1.19] |
| **Sex of household head** |  |  |  |  |
| Male |  |  | [1.00,1.00] | [1.00,1.00] |
| Female |  |  | 0.864 | 0.873 |
|  |  |  | [0.70,1.06] | [0.70,1.09] |
| **Random effects** |  |  |  |  |
| PSU variance (95% CI) |  | 0.57(0.42-0.75) | 0.38(0.244-0.593) | 0.36(0.22-0.58) |
| ICC | 0.103 | 0.107 | 0.103 | 0.100 |
| Wald chi-square and p-value | Ref | 41.52(p<0.001) | 10.38(0.109) | 53.84(p<0.001) |
| LR Test | 59.79 | 58.14 | 60.62 | 51.09 |
| **Model fitness** |  |  |  |  |
| Log-likelihood | -1453.199 | -1431.811 | -1447.916 | -1425.406 |
| AIC | 2910.398 | 2889.623 | 2911.831 | 2888.812 |
| N | 2161 | 2161 | 2161 | 2161 |

Exponentiated coefficients; 95% confidence intervals in brackets

^*^ *p* < 0.05, ^**^ *p* < 0.01, ^***^ *p* < 0.001 , [1.00,1.00]=Ref; PSU=Primary Sampling Unit

ICC = Intra-Class Correlation; LR Test= Likelihood ratio Test; AIC = Akaike’s Information Criterion

Model 0 is the null model, a baseline model without any determinant variable

Model 1 = Individual level variables

Model 2 = Community level variables

Model 3 is the final model adjusted for individual and household/community level variables

**Table S3: Multi-level regression analysis on predictors of optimal IPTp-SP in last Pregnancy among women in Kenya**

|  | Model 0 | **Model 1** | **Model 2** | **Model 3** |
| --- | --- | --- | --- | --- |
| **Variable** |  | **aOR[95%CI]** | **aOR[95%CI]** | **aOR[95%CI]** |
| **Age** |  |  |  |  |
| 15-19 |  | [1.00,1.00] |  | [1.00,1.00] |
| 20-24 |  | 0.659 |  | 0.661 |
|  |  | [0.43,1.02] |  | [0.43,1.02] |
| 25-29 |  | 0.653 |  | 0.657 |
|  |  | [0.42,1.01] |  | [0.42,1.02] |
| 30-34 |  | 0.636 |  | 0.643 |
|  |  | [0.39,1.03] |  | [0.40,1.04] |
| 35-39 |  | 0.808 |  | 0.817 |
|  |  | [0.48,1.37] |  | [0.48,1.39] |
| 40-44 |  | 0.533 |  | 0.543 |
|  |  | [0.27,1.04] |  | [0.28,1.06] |
| 45-49 |  | 0.954 |  | 0.987 |
|  |  | [0.33,2.76] |  | [0.34,2.87] |
| **Education** |  |  |  |  |
| No formal education |  | [1.00,1.00] |  | [1.00,1.00] |
| Primary |  | 1.018 |  | 1.087 |
|  |  | [0.72,1.45] |  | [0.76,1.56] |
| SHS/Tertiary |  | 0.896 |  | 0.972 |
|  |  | [0.60,1.35] |  | [0.64,1.49] |
| **Parity** |  |  |  |  |
| 1-3 |  | 1.308 |  | 1.322 |
|  |  | [0.98,1.75] |  | [0.99,1.77] |
| 4 or more |  | [1.00,1.00] |  | [1.00,1.00] |
|  |  |  |  |  |
| **Exposure to malaria information on Radio** |  |  |  |  |
| No |  | [1.00,1.00] |  | [1.00,1.00] |
| Yes |  | 0.860 |  | 0.888 |
|  |  | [0.68,1.09] |  | [0.69,1.15] |
| **Exposure to malaria information on TV** |  |  |  |  |
| No |  | [1.00,1.00] |  | [1.00,1.00] |
| Yes |  | 1.020 |  | 1.075 |
|  |  | [0.77,1.35] |  | [0.70,1.66] |
| **Place of Residence** |  |  |  |  |
| Urban |  |  | [1.00,1.00] | [1.00,1.00] |
| Rural |  |  | 0.811 | 0.828 |
|  |  |  | [0.58,1.14] | [0.59,1.17] |
| **Wealth** |  |  |  |  |
| Poorest |  |  | [1.00,1.00] | [1.00,1.00] |
| Poorer |  |  | 0.734 | 0.715 |
|  |  |  | [0.53,1.01] | [0.51,1.01] |
| Middle |  |  | 0.944 | 0.929 |
|  |  |  | [0.67,1.32] | [0.64,1.35] |
| Richer |  |  | 0.751 | 0.744 |
|  |  |  | [0.52,1.08] | [0.45,1.22] |
| Richest |  |  | 0.766 | 0.739 |
|  |  |  | [0.51,1.15] | [0.39,1.39] |
| **Sex of household head** | |  |  |  |
| Male |  |  | [1.00,1.00] | [1.00,1.00] |
| Female |  |  | 0.963 | 0.949 |
|  |  |  | [0.77,1.21] | [0.75,1.20] |
| pseudo *R*^2^ |  |  |  |  |
| **Random effects** |  |  |  |  |
| PSU variance (95% CI) | 0.85(0.59-1.24) | 0.85(0.58-1.24) | 0.82(0.56-1.20) | 0.83(0.56-1.21) |
| ICC | 0.206 | 0.205 | 0.200 | 0.202 |
| Wald chi-square and p-value | Ref | 14.92(0.186) | 105.29 | 20.84(0.233) |
| LR Test | 60.63 | 107.29 | 219.04 | 103.68 |
| **Model fitness** |  |  |  |  |
| Log-likelihood | -1271.925 | --1264.453 | -1268.77 | -1261.411 |
| AIC | 2547.849 | 2554.907 | 2553.541 | 2560.822 |
| N | 2232 | 2232 | 2232 | 2232 |

Exponentiated coefficients; 95% confidence intervals in brackets

^*^ *p* < 0.05, ^**^ *p* < 0.01, ^***^ *p* < 0.001 , [1.00,1.00]=Ref; PSU=Primary Sampling Unit

ICC = Intra-Class Correlation; LR Test= Likelihood ratio Test; AIC = Akaike’s Information Criterion

Model 0 is the null model, a baseline model without any determinant variable

Model 1 = Individual level variables

Model 2 = Community level variables

Model 3 is the final model adjusted for individual and household/community level variables

**Table S4: Multi-level regression analysis on predictors of optimal IPTp-SP in last Pregnancy among women in Liberia**

|  | **Model 0** | **Model 1** | **Model 2** | **Model 3** |  |  |
| --- | --- | --- | --- | --- | --- | --- |
| **Variable** |  | **aOR[95%CI]** | **aOR[95%CI]** | **aOR[95%CI]** |  |  |
| **Age** |  |  |  |  |  |  |
| 15-19 |  | [1.00,1.00] |  | [1.00,1.00] |  |  |
| 20-24 |  | 1.084 |  | 1.070 |  |  |
|  |  | [0.75,1.56] |  | [0.74,1.54] |  |  |
| 25-29 |  | 0.896 |  | 0.877 |  |  |
|  |  | [0.60,1.33] |  | [0.59,1.31] |  |  |
| 30-34 |  | 1.066 |  | 1.051 |  |  |
|  |  | [0.69,1.65] |  | [0.67,1.64] |  |  |
| 35-39 |  | 1.366 |  | 1.346 |  |  |
|  |  | [0.84,2.22] |  | [0.83,2.19] |  |  |
| 40-44 |  | 0.980 |  | 0.974 |  |  |
|  |  | [0.55,1.75] |  | [0.54,1.75] |  |  |
| 45-49 |  | 1.340 |  | 1.254 |  |  |
|  |  | [0.59,3.02] |  | [0.55,2.83] |  |  |
| **Education** |  |  |  |  |  |  |
| No formal education |  | [1.00,1.00] |  | [1.00,1.00] |  |  |
| Primary |  | 1.186 |  | 1.181 |  |  |
|  |  | [0.91,1.55] |  | [0.90,1.54] |  |  |
| SHS/Tertiary |  | 0.838 |  | 0.814 |  |  |
|  |  | [0.63,1.12] |  | [0.60,1.10] |  |  |
| **Parity** |  |  |  |  |  |  |
| 1-3 |  | 1.166 |  | 1.144 |  |  |
|  |  | [0.86,1.58] |  | [0.84,1.55] |  |  |
| 4 or more |  | [1.00,1.00] |  | [1.00,1.00] |  | [1.00,1.00] |
| **Exposure to malaria information on Radio** |  |  |  |  |  |  |
| No |  | [1.00,1.00] |  | [1.00,1.00] |  |  |
| Yes |  | 0.896 |  | 0.826 |  |  |
|  |  | [0.72,1.11] |  | [0.66,1.04] |  |  |
| **Exposure to malaria information on TV** |  |  |  |  |  |  |
| No |  | [1.00,1.00] |  | [1.00,1.00] |  |  |
| Yes |  | 0.737 |  | 0.632 |  |  |
|  |  | [0.52,1.05] |  | [0.39,1.01] |  |  |
| **Place of Residence** |  |  |  |  |  |  |
| Urban |  |  | [1.00,1.00] | [1.00,1.00] |  |  |
| Rural |  |  | 1.393 | 1.408 |  |  |
|  |  |  | [0.96,2.03] | [0.96,2.06] |  |  |
| **Wealth** |  |  |  |  |  |  |
| Poorest |  |  | [1.00,1.00] | [1.00,1.00] |  |  |
| Poorer |  |  | 1.375^*^ | 1.446^*^ |  |  |
|  |  |  | [1.00,1.88] | [1.05,1.99] |  |  |
| Middle |  |  | 1.426^*^ | 1.629^**^ |  |  |
|  |  |  | [1.01,2.00] | [1.14,2.32] |  |  |
| Richer |  |  | 1.323 | 1.716^*^ |  |  |
|  |  |  | [0.84,2.08] | [1.05,2.79] |  |  |
| Richest |  |  | 1.132 | 2.129^*^ |  |  |
|  |  |  | [0.68,1.88] | [1.07,4.22] |  |  |
| **Sex of household head** | |  |  |  |  |  |
| Male |  |  | [1.00,1.00] | [1.00,1.00] |  |  |
| Female |  |  | 1.103 | 1.073 |  |  |
|  |  |  | [0.88,1.39] | [0.85,1.36] |  |  |
| **Random effects** |  |  |  |  |  |  |
| PSU variance (95% CI) | 0.49(0.31-0.78) | 0.46(0.28-0.75) | 0.44(0.27-0.73) | 0.44(0.27-0.73) |  |  |
| ICC | 0.130 | 0.123 |  |  |  |  |
| Wald chi-square and p-value | Ref | 17.43(0.098) | 9.64(0.141) | 27.47(0.05) |  |  |
| LR Test | 490.76 | 54.11 | 50.44 | 49.80 |  |  |
| **Model fitness** |  |  |  |  |  |  |
| Log-likelihood | -1193.944 | -1185.085 | -1189.178 | -1179.968 |  |  |
| AIC | 2391.889 | 2396.17 | 2394.356 | 2397.935 |  |  |
| N | 2126 | 2126 | 2126 | 2126 |  |  |

Exponentiated coefficients; 95% confidence intervals in brackets

^*^ *p* < 0.05, ^**^ *p* < 0.01, ^***^ *p* < 0.001 , [1.00,1.00]=Ref; PSU=Primary Sampling Unit

ICC = Intra-Class Correlation; LR Test= Likelihood ratio Test; AIC = Akaike’s Information Criterion

Model 0 is the null model, a baseline model without any determinant variable

Model 1 = Individual level variables

Model 2 = Community level variables

Model 3 is the final model adjusted for individual and household/community level variables

**Table S5: Multi-level regression analysis on predictors of optimal IPTp-SP in last Pregnancy among women in Madagascar**

|  | Model 0 | **Model 1** | **Model 2** | **Model 3** |
| --- | --- | --- | --- | --- |
| **Variable** |  | **aOR[95%CI]** | **aOR[95%CI]** | **aOR[95%CI]** |
| **Age** |  |  |  |  |
| 15-19 |  | [1.00,1.00] |  | [1.00,1.00] |
| 20-24 |  | 0.839 |  | 0.845 |
|  |  | [0.60,1.18] |  | [0.60,1.19] |
| 25-29 |  | 1.019 |  | 1.015 |
|  |  | [0.72,1.44] |  | [0.72,1.44] |
| 30-34 |  | 1.173 |  | 1.162 |
|  |  | [0.81,1.70] |  | [0.80,1.69] |
| 35-39 |  | 1.260 |  | 1.239 |
|  |  | [0.84,1.90] |  | [0.82,1.87] |
| 40-44 |  | 1.216 |  | 1.180 |
|  |  | [0.74,1.99] |  | [0.72,1.94] |
| 45-49 |  | 0.691 |  | 0.681 |
|  |  | [0.33,1.45] |  | [0.32,1.43] |
| **Education** |  |  |  |  |
| No formal education |  | [1.00,1.00] |  | [1.00,1.00] |
| Primary |  | 1.231 |  | 1.221 |
|  |  | [0.95,1.59] |  | [0.94,1.58] |
| SHS/Tertiary |  | 1.407^*^ |  | 1.344 |
|  |  | [1.04,1.91] |  | [0.98,1.85] |
| **Parity** |  |  |  |  |
| 1-3 |  | 0.850 |  | 0.828 |
|  |  | [0.66,1.09] |  | [0.64,1.07] |
| 4 or more |  | [1.00,1.00] |  | [1.00,1.00] |
| **Exposure to malaria information on Radio** |  |  |  |  |
| No |  | [1.00,1.00] |  | [1.00,1.00] |
| Yes |  | 1.062 |  | 1.090 |
|  |  | [0.86,1.31] |  | [0.87,1.36] |
| **Exposure to malaria information on TV** |  |  |  |  |
| No |  | [1.00,1.00] |  | [1.00,1.00] |
| Yes |  | 1.297 |  | 1.207 |
|  |  | [0.95,1.76] |  | [0.80,1.81] |
| **Place of Residence** |  |  |  |  |
| Urban |  |  | [1.00,1.00] | [1.00,1.00] |
| Rural |  |  | 0.821 | 0.824 |
|  |  |  | [0.55,1.23] | [0.55,1.24] |
| **Wealth** |  |  |  |  |
| Poorest |  |  | [1.00,1.00] | [1.00,1.00] |
| Poorer |  |  | 1.150 | 1.115 |
|  |  |  | [0.85,1.55] | [0.82,1.52] |
| Middle |  |  | 0.931 | 0.861 |
|  |  |  | [0.67,1.29] | [0.61,1.21] |
| Richer |  |  | 1.324 | 1.183 |
|  |  |  | [0.95,1.84] | [0.83,1.69] |
| Richest |  |  | 1.397 | 1.057 |
|  |  |  | [0.95,2.06] | [0.63,1.79] |
| **Sex of household head** | |  |  |  |
| Male |  |  | [1.00,1.00] | [1.00,1.00] |
| Female |  |  | 1.364^**^ | 1.378^**^ |
|  |  |  | [1.10,1.69] | [1.11,1.71] |
| **Random effects** |  |  |  |  |
| PSU variance (95% CI) | 0.80(0.56-1.14) | 0.78(0.54-1.11) | 0.73(0.51-1.06) | 0.74(0.51-1.07) |
| ICC | 0.196 | 0.191 | 0.183 | 0.184 |
| Wald chi-square and p-value | Ref | 25.50(0.007) | 19.81(0.003) | 39.52(0.001) |
| LR Test | 99.52 | 94.12 | 88.42 | 86.90 |
| **Model fitness** |  |  |  |  |
| Log-likelihood | -1668.8447 | -1656.0167 | -1659.081 | -1648.962 |
| AIC | 3341.689 | 3338.033 | 3334.163 | 3335.925 |
| N | 5092 | 5092 | 5092 | 5092 |

Exponentiated coefficients; 95% confidence intervals in brackets

^*^ *p* < 0.05, ^**^ *p* < 0.01, ^***^ *p* < 0.001 , [1.00,1.00]=Ref; PSU=Primary Sampling Unit

ICC = Intra-Class Correlation; LR Test= Likelihood ratio Test; AIC = Akaike’s Information Criterion

Model 0 is the null model, a baseline model without any determinant variable

Model 1 = Individual level variables

Model 2 = Community level variables

Model 3 is the final model adjusted for individual and household/community level variables

**Table S6: Multi-level regression analysis on predictors of optimal IPTp-SP in last Pregnancy among women in Malawi**

|  | Model 0 | **Model 1** | **Model 2** | **Model 3** |
| --- | --- | --- | --- | --- |
| **Variable** |  | **aOR[95%CI]** | **aOR[95%CI]** | **aOR[95%CI]** |
| **Age** |  |  |  |  |
| 15-19 |  | [1.00,1.00] |  | [1.00,1.00] |
| 20-24 |  | 1.042 |  | 1.029 |
|  |  | [0.72,1.50] |  | [0.71,1.48] |
| 25-29 |  | 0.745 |  | 0.740 |
|  |  | [0.51,1.09] |  | [0.51,1.08] |
| 30-34 |  | 0.778 |  | 0.778 |
|  |  | [0.51,1.19] |  | [0.51,1.20] |
| 35-39 |  | 0.657 |  | 0.660 |
|  |  | [0.40,1.09] |  | [0.40,1.10] |
| 40-44 |  | 0.509^*^ |  | 0.498^*^ |
|  |  | [0.26,0.99] |  | [0.25,0.97] |
| 45-49 |  | 0.253^*^ |  | 0.248^*^ |
|  |  | [0.08,0.81] |  | [0.08,0.79] |
| **Education** |  |  |  |  |
| No formal education |  | [1.00,1.00] |  | [1.00,1.00] |
| Primary |  | 0.842 |  | 0.851 |
|  |  | [0.59,1.21] |  | [0.59,1.23] |
| SHS/Tertiary |  | 1.046 |  | 1.170 |
|  |  | [0.69,1.58] |  | [0.76,1.79] |
| **Parity** |  |  |  |  |
| 1-3 |  | 1.035 |  | 1.053 |
|  |  | [0.77,1.39] |  | [0.78,1.42] |
| 4 or more |  | [1.00,1.00] |  | [1.00,1.00] |
| **Exposure to malaria information on Radio** |  |  |  |  |
| No |  | [1.00,1.00] |  | [1.00,1.00] |
| Yes |  | 1.065 |  | 1.092 |
|  |  | [0.85,1.34] |  | [0.85,1.40] |
| **Exposure to malaria information on TV** |  |  |  |  |
| No |  | [1.00,1.00] |  | [1.00,1.00] |
| Yes |  | 0.764 |  | 0.866 |
|  |  | [0.56,1.04] |  | [0.61,1.23] |
| **Place of Residence** |  |  |  |  |
| Urban |  |  | [1.00,1.00] | [1.00,1.00] |
| Rural |  |  | 1.127 | 1.221 |
|  |  |  | [0.81,1.56] | [0.87,1.71] |
| **Wealth** |  |  |  |  |
| Poorest |  |  | [1.00,1.00] | [1.00,1.00] |
| Poorer |  |  | 1.088 | 1.120 |
|  |  |  | [0.76,1.55] | [0.78,1.61] |
| Middle |  |  | 1.248 | 1.285 |
|  |  |  | [0.87,1.79] | [0.88,1.87] |
| Richer |  |  | 0.953 | 0.922 |
|  |  |  | [0.67,1.36] | [0.63,1.35] |
| Richest |  |  | 0.922 | 0.899 |
|  |  |  | [0.62,1.38] | [0.56,1.45] |
| **Sex of household head** | |  |  |  |
| Male |  |  | [1.00,1.00] | [1.00,1.00] |
| Female |  |  | 0.897 | 0.889 |
|  |  |  | [0.70,1.15] | [0.69,1.15] |
| **Random effects** |  |  |  |  |
| PSU variance (95% CI) | 0.16(0.07-0.35) | 0.17(0.07-0.36) | 0.15(0.06-0.34) | 0.16(0.07-0.36) |
| ICC | 0.046 | 0.04 | 0.04 | 0.04 |
| Wald chi-square and p-value | Ref | 25.15(0.009) | 7.53(0.27) | 34.56(0.007) |
| LR Test | 10.78 | 11.61 | 9.86 | 11.07 |
| **Model fitness** |  |  |  |  |
| Log-likelihood | -1210.165 | -1196.737 | -1206.393 | -1191.6814 |
| AIC | 2424.331 | 2419.474 | 2428.786 | 2421.363 |
| N | 2102 | 2102 | 2102 | 2102 |

Exponentiated coefficients; 95% confidence intervals in brackets

^*^ *p* < 0.05, ^**^ *p* < 0.01, ^***^ *p* < 0.001 , [1.00,1.00]=Ref; PSU=Primary Sampling Unit

ICC = Intra-Class Correlation; LR Test= Likelihood ratio Test; AIC = Akaike’s Information Criterion

Model 0 is the null model, a baseline model without any determinant variable

Model 1 = Individual level variables

Model 2 = Community level variables

Model 3 is the final model adjusted for individual and household/community level variables

**Table S7: Multi-level regression analysis on predictors of optimal IPTp-SP in last Pregnancy among women in Mali**

|  | Model 0 | Model 1 | Model 2 | Model 3 |
| --- | --- | --- | --- | --- |
| **Variable** |  | aOR[95%CI] | aOR[95%CI] | aOR[95%CI] |
| **Age** |  |  |  |  |
| 15-19 |  | [1.00,1.00] |  | [1.00,1.00] |
| 20-24 |  | 1.268 |  | 1.263 |
|  |  | [0.96,1.67] |  | [0.96,1.67] |
| 25-29 |  | 1.439^*^ |  | 1.430^*^ |
|  |  | [1.07,1.93] |  | [1.06,1.92] |
| 30-34 |  | 1.892^***^ |  | 1.879^***^ |
|  |  | [1.38,2.60] |  | [1.36,2.59] |
| 35-39 |  | 1.513^*^ |  | 1.493^*^ |
|  |  | [1.07,2.14] |  | [1.05,2.11] |
| 40-44 |  | 1.951^***^ |  | 1.926^***^ |
|  |  | [1.32,2.87] |  | [1.31,2.84] |
| 45-49 |  | 1.928^*^ |  | 1.936^*^ |
|  |  | [1.16,3.20] |  | [1.17,3.22] |
| **Education** |  |  |  |  |
| No formal education |  | [1.00,1.00] |  | [1.00,1.00] |
| Primary |  | 1.185 |  | 1.183 |
|  |  | [0.96,1.46] |  | [0.96,1.46] |
| SHS/Tertiary |  | 1.366^**^ |  | 1.334^*^ |
|  |  | [1.08,1.72] |  | [1.05,1.69] |
| **Parity** |  |  |  |  |
| 1-3 |  | 1.086 |  | 1.081 |
|  |  | [0.90,1.32] |  | [0.89,1.31] |
| 4 or more |  | [1.00,1.00] |  | [1.00,1.00] |
| **Exposure to malaria information on Radio** |  |  |  |  |
| No |  | [1.00,1.00] |  | [1.00,1.00] |
| Yes |  | 1.285^**^ |  | 1.285^**^ |
|  |  | [1.08,1.52] |  | [1.08,1.52] |
| **Exposure to malaria information on TV** |  |  |  |  |
| No |  | [1.00,1.00] |  | [1.00,1.00] |
| Yes |  | 1.083 |  | 1.079 |
|  |  | [0.92,1.27] |  | [0.91,1.28] |
| **Place of Residence** |  |  |  |  |
| Urban |  |  | [1.00,1.00] | [1.00,1.00] |
| Rural |  |  | 0.743 | 0.782 |
|  |  |  | [0.52,1.07] | [0.54,1.12] |
| **Wealth** |  |  |  |  |
| Poorest |  |  | [1.00,1.00] | [1.00,1.00] |
| Poorer |  |  | 0.969 | 0.961 |
|  |  |  | [0.77,1.21] | [0.77,1.20] |
| Middle |  |  | 0.762^*^ | 0.741^*^ |
|  |  |  | [0.60,0.97] | [0.58,0.95] |
| Richer |  |  | 0.884 | 0.844 |
|  |  |  | [0.67,1.16] | [0.64,1.11] |
| Richest |  |  | 0.955 | 0.817 |
|  |  |  | [0.64,1.41] | [0.54,1.23] |
| **Sex of household head** | |  |  |  |
| Male |  |  | [1.00,1.00] | [1.00,1.00] |
| Female |  |  | 0.889 | 0.905 |
|  |  |  | [0.61,1.30] | [0.62,1.33] |
| **Random effects** |  |  |  |  |
| PSU variance (95% CI) | 0.31(0.21-0.46) | 0.29(0.19-0.44) | 0.32(0.21-0.47) | 0.31(0.21-0.46) |
| ICC | 0.088 | 0.08 | 0.09 | 0.09 |
| Wald chi-square and p-value | Ref | 42.84(<0.001) | 13.13(0.041) | 51.78(<0.001)) |
| LR Test | 97.83 | 85.83 | 92.39 | 88.73 |
| **Model fitness** |  |  |  |  |
| Log-likelihood | -2642.352 | -2620.647 | -2635.629 | -2615.768 |
| AIC | 5288.704 | 5267.293 | 5287.258 | 5269.535 |
| N | 5054 | 5054 | 5054 | 5054 |

Exponentiated coefficients; 95% confidence intervals in brackets

^*^ *p* < 0.05, ^**^ *p* < 0.01, ^***^ *p* < 0.001 , [1.00,1.00]=Ref; PSU=Primary Sampling Unit

ICC = Intra-Class Correlation; LR Test= Likelihood ratio Test; AIC = Akaike’s Information Criterion

Model 0 is the null model, a baseline model without any determinant variable

Model 1 = Individual level variables

Model 2 = Community level variables

Model 3 is the final model adjusted for individual and household/community level variables

**Table S8: Multi-level regression analysis on predictors of optimal IPTp-SP in last Pregnancy among women in Mozambique**

|  | **Model 0** | **Model 1** | **Model 2** | **Model 3** |
| --- | --- | --- | --- | --- |
| **Variable** |  | **aOR[95%CI]** | **aOR[95%CI]** | **aOR[95%CI]** |
| **Age** |  |  |  |  |
| 15-19 |  | [1.00,1.00] |  | [1.00,1.00] |
| 20-24 |  | 1.167 |  | 1.172 |
|  |  | [0.91,1.50] |  | [0.91,1.51] |
| 25-29 |  | 1.112 |  | 1.094 |
|  |  | [0.84,1.47] |  | [0.83,1.45] |
| 30-34 |  | 0.976 |  | 0.935 |
|  |  | [0.71,1.35] |  | [0.68,1.29] |
| 35-39 |  | 0.931 |  | 0.876 |
|  |  | [0.65,1.34] |  | [0.61,1.27] |
| 40-44 |  | 0.822 |  | 0.785 |
|  |  | [0.54,1.24] |  | [0.52,1.19] |
| 40-49 |  | 1.007 |  | 0.970 |
|  |  | [0.57,1.77] |  | [0.55,1.71] |
| **Education** |  |  |  |  |
| No formal education |  | [1.00,1.00] |  | [1.00,1.00] |
| Primary |  | 0.975 |  | 0.937 |
|  |  | [0.79,1.21] |  | [0.76,1.16] |
| SHS/Tertiary |  | 1.289 |  | 1.119 |
|  |  | [0.98,1.70] |  | [0.84,1.50] |
| **Parity** |  |  |  |  |
| 1-3 |  | 1.170 |  | 1.138 |
|  |  | [0.93,1.46] |  | [0.91,1.43] |
| 4 or more |  | [1.00,1.00] |  | [1.00,1.00] |
| **Exposure to malaria information on Radio** |  |  |  |  |
| No |  | [1.00,1.00] |  | [1.00,1.00] |
| Yes |  | 1.092 |  | 1.098 |
|  |  | [0.92,1.29] |  | [0.92,1.31] |
| **Exposure to malaria information on TV** |  |  |  |  |
| No |  | [1.00,1.00] |  | [1.00,1.00] |
| Yes |  | 1.211 |  | 0.917 |
|  |  | [0.97,1.51] |  | [0.68,1.23] |
| **Place of Residence** |  |  |  |  |
| Urban |  |  | [1.00,1.00] | [1.00,1.00] |
| Rural |  |  | 0.905 | 0.926 |
|  |  |  | [0.68,1.21] | [0.69,1.25] |
| **Wealth** |  |  | 1 | 1 |
| Poorest |  |  | [1.00,1.00] | [1.00,1.00] |
| Poorer |  |  | 1.041 | 1.000 |
|  |  |  | [0.80,1.35] | [0.77,1.30] |
| Middle |  |  | 0.981 | 0.950 |
|  |  |  | [0.75,1.29] | [0.71,1.26] |
| Richer |  |  | 1.500^**^ | 1.429^*^ |
|  |  |  | [1.12,2.01] | [1.03,1.98] |
| Richest |  |  | 1.803^***^ | 1.655^*^ |
|  |  |  | [1.27,2.56] | [1.04,2.64] |
| **Sex of household head** | |  |  |  |
| Male |  |  | [1.00,1.00] | [1.00,1.00] |
| Female |  |  | 1.122 | 1.126 |
|  |  |  | [0.95,1.33] | [0.95,1.34] |
| **Random effects** |  |  |  |  |
| PSU variance (95% CI) | 0.51(0.37-0.72) | 0.45(0.32-0.65) | 0.46(0.32-0.65) | 0.46(0.32-0.65) |
| ICC | 0.136 | 0.12 | 0.122 | 0.12 |
| Wald chi-square and p-value | Ref | 39.56(<0.001) | 30.38(<0.001) | 52.59(<0.001) |
| LR Test | 132.18 | 109.43 | 112.83 | 109.33 |
| **Model fitness** |  |  |  |  |
| Log-likelihood | -2078.097 | -2058.318 | -2062.971 | -2051.5 |
| AIC | 4160.195 | 4142.636 | 4141.941 | 4140.999 |
| PSU | 224 | 224 | 224 | 224 |
| N | 3288 | 3288 | 3288 | 3288 |

Exponentiated coefficients; 95% confidence intervals in brackets

^*^ *p* < 0.05, ^**^ *p* < 0.01, ^***^ *p* < 0.001 , [1.00,1.00]=Ref; PSU=Primary Sampling Unit

ICC = Intra-Class Correlation; LR Test= Likelihood ratio Test; AIC = Akaike’s Information Criterion

Model 0 is the null model, a baseline model without any determinant variable

Model 1 = Individual level variables

Model 2 = Community level variables

Model 3 is the final model adjusted for individual and household/community level variables

**Table S9: Multi-level regression analysis on predictors of optimal IPTp-SP in last Pregnancy among women in Nigeria**

|  | **Model 0** | **Model 1** | **Model 2** | **Model 3** |
| --- | --- | --- | --- | --- |
| **Variable** |  | **aOR[95%CI]** | **aOR[95%CI]** | **aOR[95%CI]** |
| **Age** |  |  |  |  |
| 15-19 |  | **Ref** |  | **Ref** |
| 20-24 |  | 1.045 |  | 1.027 |
|  |  | [0.71,1.53] |  | [0.70,1.50] |
| 25-29 |  | 1.201 |  | 1.188 |
|  |  | [0.82,1.77] |  | [0.81,1.75] |
| 30-34 |  | 0.924 |  | 0.901 |
|  |  | [0.61,1.40] |  | [0.59,1.36] |
| 35-39 |  | 0.941 |  | 0.914 |
|  |  | [0.60,1.47] |  | [0.58,1.43] |
| 40-44 |  | 1.185 |  | 1.135 |
|  |  | [0.71,1.97] |  | [0.68,1.89] |
| 45-49 |  | 1.186 |  | 1.139 |
|  |  | [0.61,2.32] |  | [0.58,2.23] |
| **Education** |  |  |  |  |
| No formal education |  | **Ref** |  | **Ref** |
| Primary |  | 1.422^**^ |  | 1.347^*^ |
|  |  | [1.09,1.85] |  | [1.03,1.76] |
| SHS/Tertiary |  | 1.502^**^ |  | 1.385^*^ |
|  |  | [1.16,1.94] |  | [1.06,1.81] |
| **Parity** |  |  |  |  |
| 1-3 |  | 0.878 |  | 0.865 |
|  |  | [0.71,1.08] |  | [0.70,1.07] |
| 4 or more |  | **Ref** |  | **Ref** |
| **Exposure to malaria information on Radio** |  |  |  |  |
| No |  | **Ref** |  | **Ref** |
| Yes |  | 1.061 |  | 1.049 |
|  |  | [0.87,1.29] |  | [0.86,1.28] |
| **Exposure to malaria information on TV** |  | 1 |  | 1 |
| No |  | **Ref** |  | **Ref** |
| Yes |  | 1.463^**^ |  | 1.245 |
|  |  | [1.16,1.85] |  | [0.94,1.64] |
| **Place of Residence** |  |  |  |  |
| Urban |  |  | **Ref** | **Ref** |
| Rural |  |  | 0.676^*^ | 0.679^*^ |
|  |  |  | [0.49,0.93] | [0.49,0.94] |
| **Wealth** |  |  |  |  |
| Poorest |  |  | **Ref** | **Ref** |
| Poorer |  |  | 1.516^*^ | 1.454^*^ |
|  |  |  | [1.09,2.10] | [1.05,2.02] |
| Middle |  |  | 1.774^**^ | 1.510^*^ |
|  |  |  | [1.25,2.52] | [1.04,2.19] |
| Richer |  |  | 2.289^***^ | 1.659^*^ |
|  |  |  | [1.56,3.36] | [1.05,2.61] |
| Richest |  |  | 2.125^***^ | 1.452 |
|  |  |  | [1.39,3.24] | [0.86,2.44] |
| **Sex of household head** |  |  |  |  |
| Male |  |  | **Ref** | **Ref** |
| Female |  |  | 0.846 | 0.849 |
|  |  |  | [0.61,1.17] | [0.61,1.18] |
| **Random effects** |  |  |  |  |
| PSU variance (95% CI) | 1.08(0.81-1.44) | 0.96(0.71-1.29) | 0.89(0.65-1.21)) | 0.88 (0.65-1.20) |
| ICC | 0.248 | 0.222 | 0.221 | 0.211 |
| Wald chi-square and p-value | Ref | 40.85(<0.001) | 38.31(<0.001) | 56.19(<0.001) |
| LR Test | 254.98 | 216.24 | 193.62 | 190.78 |
| **Model fitness** |  |  |  |  |
| Log-likelihood | -1926.266 | -1905.92 | -1907.509 | -1898.342 |
| AIC | 3856.532 | 3837.839 | 3831.017 | 3834.685 |
| N | 3923 | 3923 | 3923 | 3923 |

Exponentiated coefficients; 95% confidence intervals in brackets

^*^ *p* < 0.05, ^**^ *p* < 0.01, ^***^ *p* < 0.001 , [1.00,1.00]=Ref; PSU=Primary Sampling Unit

ICC = Intra-Class Correlation; LR Test= Likelihood ratio Test; AIC = Akaike’s Information Criterion

Model 0 is the null model, a baseline model without any determinant variable

Model 1 = Individual level variables

Model 2 = Community level variables

Model 3 is the final model adjusted for individual and household/community level variables

**Table S10: Multi-level regression analysis on predictors of optimal IPTp-SP in last Pregnancy among women in Sierra Leone**

|  | Model 0 | Model 1 | Model 2 | Model 3 |
| --- | --- | --- | --- | --- |
| **Variable** |  | aOR[95%CI] | aOR[95%CI] | aOR[95%CI] |
| **Age** |  |  |  |  |
| 15-19 |  | [1.00,1.00] |  | [1.00,1.00] |
| 20-24 |  | 0.961 |  | 0.998 |
|  |  | [0.74,1.24] |  | [0.77,1.29] |
| 25-29 |  | 0.788 |  | 0.825 |
|  |  | [0.60,1.03] |  | [0.63,1.08] |
| 30-34 |  | 0.808 |  | 0.863 |
|  |  | [0.60,1.09] |  | [0.64,1.17] |
| 35-39 |  | 0.799 |  | 0.840 |
|  |  | [0.58,1.10] |  | [0.61,1.16] |
| 40-44 |  | 1.073 |  | 1.139 |
|  |  | [0.70,1.65] |  | [0.74,1.76] |
| 45-49 |  | 1.727 |  | 1.793^*^ |
|  |  | [0.99,3.03] |  | [1.02,3.14] |
| **Education** |  |  |  |  |
| No formal education |  | [1.00,1.00] |  | [1.00,1.00] |
| Primary |  | 0.918 |  | 0.932 |
|  |  | [0.74,1.13] |  | [0.75,1.15] |
| SHS/Tertiary |  | 0.779^*^ |  | 0.817 |
|  |  | [0.64,0.95] |  | [0.67,1.00] |
|  |  |  |  |  |
| **Parity** |  | 1.030 |  | 1.051 |
| 1-3 |  | [0.86,1.23] |  | [0.88,1.26] |
| 4 or more |  | [1.00,1.00] |  | [1.00,1.00] |
| **Exposure to malaria information on Radio** |  |  |  |  |
| No |  | [1.00,1.00] |  | [1.00,1.00] |
| Yes |  | 0.968 |  | 0.979 |
|  |  | [0.83,1.12] |  | [0.84,1.15] |
| **Exposure to malaria information on TV** |  |  |  |  |
| No |  | [1.00,1.00] |  | [1.00,1.00] |
| Yes |  | 0.734^*^ |  | 1.167 |
|  |  | [0.55,0.98] |  | [0.80,1.71] |
| **Place of Residence** |  |  |  |  |
| Urban |  |  | [1.00,1.00] | [1.00,1.00] |
| Rural |  |  | 1.149 | 1.137 |
|  |  |  | [0.84,1.58] | [0.83,1.56] |
| **Wealth** |  |  |  |  |
| Poorest |  |  | [1.00,1.00] | [1.00,1.00] |
| Poorer |  |  | 1.072 | 1.091 |
|  |  |  | [0.87,1.31] | [0.88,1.35] |
| Middle |  |  | 1.115 | 1.141 |
|  |  |  | [0.90,1.39] | [0.91,1.43] |
| Richer |  |  | 0.800 | 0.823 |
|  |  |  | [0.60,1.06] | [0.61,1.11] |
| Richest |  |  | 0.531^**^ | 0.515^**^ |
|  |  |  | [0.36,0.78] | [0.31,0.85] |
| **Sex of household head** | |  |  |  |
| Male |  |  | [1.00,1.00] | [1.00,1.00] |
| Female |  |  | 0.944 | 0.942 |
|  |  |  | [0.79,1.12] | [0.79,1.12] |
| **Random effects** |  |  |  |  |
| PSU variance (95% CI) | 0.85(0.66-1.106) | 0.82(0.62-1.07) | 0.77(0.59-1.00) | 0.77(0.59-1.01) |
| ICC | 0.206 | 0.199 | 0.189 | 0.191 |
| Wald chi-square and p-value | Ref | 27.58(0.0038) | 28.41(0.001) | 46.02(0.001) |
| LR Test | 286.49 | 264.76 | 251.05 | 250.97 |
| **Model fitness** |  |  |  |  |
| Log-likelihood | -2689.864 | -2676.056 | -2675.563 | -2666.596 |
| AIC | 5383.728 | 5378.112 | 5367.127 | 5371.192 |
| N | 4457 | 4457 | 4457 | 4457 |

Exponentiated coefficients; 95% confidence intervals in brackets

^*^ *p* < 0.05, ^**^ *p* < 0.01, ^***^ *p* < 0.001 , [1.00,1.00]=Ref; PSU=Primary Sampling Unit

ICC = Intra-Class Correlation; LR Test= Likelihood ratio Test; AIC = Akaike’s Information Criterion

Model 0 is the null model, a baseline model without any determinant variable

Model 1 = Individual level variables

Model 2 = Community level variables

Model 3 is the final model adjusted for individual and household/community level variables

**Table S11: Multi-level regression analysis on predictors of optimal IPTp-SP in last Pregnancy among women in** Tanzania

|  | **Model0** | **Model 1** | **Model 2** | **Model 3** |
| --- | --- | --- | --- | --- |
| **Variable** |  | **aOR[95%CI]** | **aOR[95%CI]** | **aOR[95%CI]** |
| **Age** |  |  |  |  |
| 15-19 |  | [1.00,1.00] |  | [1.00,1.00] |
| 20-24 |  | 0.817 |  | 0.808 |
|  |  | [0.62,1.08] |  | [0.61,1.07] |
| 25-29 |  | 0.808 |  | 0.793 |
|  |  | [0.60,1.09] |  | [0.59,1.07] |
| 30-34 |  | 1.035 |  | 1.005 |
|  |  | [0.75,1.44] |  | [0.72,1.40] |
| 35-39 |  | 0.967 |  | 0.937 |
|  |  | [0.67,1.39] |  | [0.65,1.35] |
| 40-44 |  | 0.851 |  | 0.825 |
|  |  | [0.56,1.29] |  | [0.54,1.25] |
| 45-49 |  | 0.708 |  | 0.693 |
|  |  | [0.37,1.35] |  | [0.36,1.32] |
| **Education** |  |  |  |  |
| No formal education |  | [1.00,1.00] |  | [1.00,1.00] |
| Primary |  | 1.385^**^ |  | 1.371^**^ |
|  |  | [1.13,1.70] |  | [1.12,1.69] |
| SHS/Tertiary |  | 1.466^**^ |  | 1.386^*^ |
|  |  | [1.11,1.94] |  | [1.04,1.85] |
|  |  |  |  |  |
| **Parity** |  | 1.348^**^ |  | 1.325^*^ |
| 1-3 |  | [1.09,1.67] |  | [1.07,1.65] |
| 4 or more |  | [1.00,1.00] |  | [1.00,1.00] |
| **Exposure to malaria information on Radio** |  |  |  |  |
| No |  | [1.00,1.00] |  | [1.00,1.00] |
| Yes |  | 1.210^*^ |  | 1.180 |
|  |  | [1.03,1.42] |  | [1.00,1.40] |
| **Exposure to malaria information on TV** |  |  |  |  |
| No |  | [1.00,1.00] |  | [1.00,1.00] |
| Yes |  | 0.895 |  | 0.718^*^ |
|  |  | [0.72,1.11] |  | [0.53,0.96] |
| **Place of Residence** |  |  |  |  |
| Urban |  |  | [1.00,1.00] | [1.00,1.00] |
| Rural |  |  | 0.957 | 0.962 |
|  |  |  | [0.72,1.26] | [0.73,1.27] |
| **Wealth** |  |  |  |  |
| Poorest |  |  | [1.00,1.00] | [1.00,1.00] |
| Poorer |  |  | 1.056 | 0.992 |
|  |  |  | [0.84,1.33] | [0.78,1.26] |
| Middle |  |  | 1.084 | 0.980 |
|  |  |  | [0.85,1.38] | [0.76,1.26] |
| Richer |  |  | 1.110 | 1.023 |
|  |  |  | [0.85,1.45] | [0.77,1.37] |
| Richest |  |  | 1.422^*^ | 1.441 |
|  |  |  | [1.04,1.95] | [0.93,2.24] |
| **Sex of household head** | |  |  |  |
| Male |  |  | [1.00,1.00] | [1.00,1.00] |
| Female |  |  | 0.943 | 0.933 |
|  |  |  | [0.78,1.14] | [0.77,1.14] |
| **Random effects** |  |  |  |  |
| PSU variance (95% CI) | 0.601(0.44-0.822) | 0.55(0.40-0.76) | 0.600(0.44-0.815) | 0.55(0.41-0.77) |
| ICC | 0.156 | 0.144 | 0.154 | 0.145 |
| Wald chi-square and p-value | Ref | 39.17(<0.001) | 8.51(0.20) | 45.03(<0.001) |
| LR Test | 132.82 | 114.44 | 129.40 | 114.84 |
| **Model fitness** |  |  |  |  |
| Log-likelihood | -2471.612 | -2451.553 | -2467.403 | -2448.644 |
| AIC | 4947.223 | 4929.107 | 4950.806 | 4935.289 |
| N | 5036 | 5036 | 5036 | 5036 |

Exponentiated coefficients; 95% confidence intervals in brackets

^*^ *p* < 0.05, ^**^ *p* < 0.01, ^***^ *p* < 0.001 , [1.00,1.00]=Ref; PSU=Primary Sampling Unit

ICC = Intra-Class Correlation; LR Test= Likelihood ratio Test; AIC = Akaike’s Information Criterion

Model 0 is the null model, a baseline model without any determinant variable

Model 1 = Individual level variables

Model 2 = Community level variables

Model 3 is the final model adjusted for individual and household/community level variables

**Table S12: Multi-level regression analysis on predictors of optimal IPTp-SP in last Pregnancy among women in** Uganda

|  | **Model0** | **Model 1** | **Model 2** | **Model 3** |
| --- | --- | --- | --- | --- |
| **Variable** |  | **aOR[95%CI]** | **aOR[95%CI]** | **aOR[95%CI]** |
| **Age** |  |  |  |  |
| 15-19 |  | [1.00,1.00] |  | [1.00,1.00] |
| 20-24 |  | 1.172 |  | 1.168 |
|  |  | [0.90,1.52] |  | [0.90,1.52] |
| 25-29 |  | 1.049 |  | 1.045 |
|  |  | [0.79,1.38] |  | [0.79,1.38] |
| 30-34 |  | 1.092 |  | 1.092 |
|  |  | [0.80,1.48] |  | [0.80,1.48] |
| 35-39 |  | 1.024 |  | 1.025 |
|  |  | [0.73,1.43] |  | [0.73,1.44] |
| 40-44 |  | 1.100 |  | 1.103 |
|  |  | [0.75,1.62] |  | [0.75,1.62] |
| 45-49 |  | 1.181 |  | 1.185 |
|  |  | [0.70,1.99] |  | [0.70,2.00] |
| **Education** |  |  |  |  |
| No formal education |  | [1.00,1.00] |  | [1.00,1.00] |
| Primary |  | 1.035 |  | 1.020 |
|  |  | [0.86,1.25] |  | [0.85,1.23] |
| SHS/Tertiary |  | 1.051 |  | 1.023 |
|  |  | [0.84,1.32] |  | [0.80,1.30] |
| **Parity** |  |  |  |  |
| 1-3 |  | 1.291^**^ |  | 1.297^**^ |
|  |  | [1.08,1.55] |  | [1.08,1.55] |
| 4 or more |  | [1.00,1.00] |  | [1.00,1.00] |
| **Exposure to malaria information on Radio** |  |  |  |  |
| No |  | [1.00,1.00] |  | [1.00,1.00] |
| Yes |  | 1.097 |  | 1.066 |
|  |  | [0.96,1.25] |  | [0.92,1.23] |
| **Exposure to malaria information on TV** |  |  |  |  |
| No |  | [1.00,1.00] |  | [1.00,1.00] |
| Yes |  | 1.039 |  | 0.981 |
|  |  | [0.85,1.27] |  | [0.74,1.30] |
| **Place of Residence** |  |  |  |  |
| Urban |  |  | [1.00,1.00] | [1.00,1.00] |
| Rural |  |  | 1.013 | 1.018 |
|  |  |  | [0.82,1.26] | [0.82,1.26] |
| **Wealth** |  |  |  |  |
| Poorest |  |  | [1.00,1.00] | [1.00,1.00] |
| Poorer |  |  | 1.062 | 1.040 |
|  |  |  | [0.88,1.28] | [0.86,1.26] |
| Middle |  |  | 1.042 | 1.007 |
|  |  |  | [0.85,1.28] | [0.81,1.25] |
| Richer |  |  | 1.127 | 1.082 |
|  |  |  | [0.91,1.40] | [0.85,1.38] |
| Richest |  |  | 1.208 | 1.128 |
|  |  |  | [0.94,1.55] | [0.79,1.61] |
| **Sex of household head** | |  |  |  |
| Male |  |  | [1.00,1.00] | [1.00,1.00] |
| Female |  |  | 0.885 | 0.896 |
|  |  |  | [0.76,1.03] | [0.77,1.05] |
| **Random effects** |  |  |  |  |
| PSU variance (95% CI) | 0.15(0.09-2.25) | 0.15(0.09-0.25) | 0.15(0.09-0.25) | 0.15(0.08-0.25) |
| ICC | 0.044 | 0.043 | 0.043 | 0.043 |
| Wald chi-square and p-value | Ref | 26.04(0.006) | 5.97(0.43) | 28.66(0.04) |
| LR Test | 28.51 | 26.96 | 27.28 | 26.69 |
| **Model fitness** |  |  |  |  |
| Log-likelihood | -2858.999 | -2845.909 | -2856.012 | -2844.564 |
| AIC | 5721.998 | 5717.819 | 5728.025 | 5727.128 |
| N | 4068 | 4068 | 4068 | 4068 |

Exponentiated coefficients; 95% confidence intervals in brackets

^*^ *p* < 0.05, ^**^ *p* < 0.01, ^***^ *p* < 0.001 , [1.00,1.00]=Ref; PSU=Primary Sampling Unit

ICC = Intra-Class Correlation; LR Test= Likelihood ratio Test; AIC = Akaike’s Information Criterion

Model 0 is the null model, a baseline model without any determinant variable

Model 1 = Individual level variables

Model 2 = Community level variables

Model 3 is the final model adjusted for individual and household/community level variables
